# Finite-difference based response surface methodology to optimize tailgate support systems in longwall coal mining

**DOI:** 10.1038/s41598-021-82104-8

**Published:** 2021-01-27

**Authors:** Satar Mahdevari, Mohammad Hayati

**Affiliations:** 1 Department of Mining Engineering, Hamedan University of Technology, Hamedan, Iran; 2 Department of Mining Engineering, Faculty of Engineering, Lorestan University, Khorramabad, Iran

**Keywords:** Engineering, Mathematics and computing

## Abstract

Designing a suitable support system is of great importance in longwall mining to ensure the safe and stable working conditions over the entire life of the mine. In high-speed mechanized longwall mining, the most vulnerable zones to failure are roof strata in the vicinity of the tailgate roadway and T-junctions. Severe roof displacements are occurred in the tailgate roadway due to the high-stress concentrations around the exposed roof span. In this respect, Response Surface Methodology (RSM) was utilized to optimize tailgate support systems in the Tabas longwall coal mine, northeast of Iran. The nine geomechanical parameters were obtained through the field and laboratory studies including density, uniaxial compressive strength, angle of internal friction, cohesion, shear strength, tensile strength, Young’s modulus, slake durability index, and rock mass rating. A design of experiment was developed through considering a Central Composite Design (CCD) on the independent variables. The 149 experiments are resulted based on the output of CCD, and were introduced to a software package of finite difference numerical method to calculate the maximum roof displacements (*d*_*max*_) in each experiment as the response of design. Therefore, the geomechanical variables are merged and consolidated into a modified quadratic equation for prediction of the *d*_*max*_. The proposed model was executed in four approaches of linear, two-factor interaction, quadratic, and cubic. The best squared correlation coefficient was obtained as 0.96. The prediction capability of the model was examined by testing on some unseen real data that were monitored at the mine. The proposed model appears to give a high goodness of fit with the accuracy of 0.90. These results indicate the accuracy and reliability of the developed model, which may be considered as a reliable tool for optimizing or redesigning the support systems in longwall tailgates. Analysis of variance (ANOVA) was performed to identify the key variables affecting the *d*_*max*_, and to recognize their pairwise interaction effects. The key parameters influencing the *d*_*max*_ are respectively found to be slake durability index, Young’s modulus, uniaxial compressive strength, and rock mass rating.

## Introduction

Designing a set of sustainable and workable support systems is vital in successfully operating at longwall coal mines. Longwall mining is classified as an underground mining method, which is associated with controlled caving of the roof strata concurrent with and essential to the conduct of mining^1^. Therefore, providing such safe and stable conditions to work within a longwall face and also in the two adjacent tunnels are necessary.

Experiences gained in mechanized longwall coal mining in recent decades show a progressive development in high-speed mining besides improvements in health and safety standards. Nonetheless, the problem of roof failure is still the major factor affecting longwall incidents^2^.

Almost all coal reserves in Iran are exploited by the longwall mining method, and many injuries or even fatalities are annually reported due to collapses resulted from unstable roof strata. The Tabas longwall mine is gradually proceeded to depth, and consequently unstable zones at the immediate roof rocks or caved zone are spreading in the vicinity of active longwall faces. This issue may adversely affect the serviceability of the longwall gate roadways that are the lifelines of a longwall panel, in which personnel, extracted coal, supplies, and fresh air are to pass.

Safety and profitability are both important in designing mechanized longwall mining, and it is not satisfactory to interrupt the mining operations due to the unstable gate roadways. However, stress redistribution around a longwall panel results in high displacements and deformations, which may lead to delays in the coal production plan^3^. Therefore, it is necessary to install the appropriate and adequate support systems for meticulously control the stability conditions of the mine.

Stability conditions in a longwall panel may be influenced by geological structures, advancement rate, in-situ and induced stresses, panel orientation, direction of mining, barrier pillar sizes, and support systems^4^. However, the critical unstable zones in a longwall panel are T-junctions; where the longwall face intersects the tailgate and headgate roadways. In this respect, Zhu et al.^5^ denoted that the greatest damage due to rockbursts may be encountered along the tailgate roadway and ahead of its T-junction (in advance of coalface). Therefore, designing the suitable support systems in such areas within underground coal mines are necessary to avoid serious destructions of the installed support systems. Consequently, longwall T-junctions are subjected to severe loadings and deformations due to approaching and passing the coalface^6^.

However, designing a reliable support system is practically an intricate and case-based procedure, which mostly relies on experience or trial and error. Although many empirical, analytical, and numerical methods were presented to control roof stability in mechanized longwall panels, the problem is even now a major concern in most underground coal mines.

Numerous techniques based on experimental, theoretical, and numerical analyses were established to address the problems of unstable T-junctions such as predicting roof strata behavior, redesigning support installation plan, and installing suitable support systems. Amongst them, the stability of the longwall tailgate is the most important ground control measure in the success of designing a longwall mine^7^.

Due to the importance of the problem, much research has been done in this field. Seedsman^8^ discussed the failure mechanisms of longwall tailgates through following the stress and failure paths in the roof strata, and presented the appropriate support systems for tailgate during face advancement. Tarrant^9^ combined empirical and analytical methods to design a stable layout for the longwall tailgate. Kang et al.^10^ performed a discrete element numerical method to better understand the mechanism of rock bolts in supporting longwall entries within soft rocks. Bai and Tu^11^ based on the field observations and numerical simulations investigated the stability conditions of the longwall drift. Mangal and Paul^12^ reviewed the mechanism of roof caving by theoretical methods to access the powered support resistance requirement in the mechanized longwall faces. Kang et al.^13^ combined the physical and numerical modeling to gain a better understanding of failure mechanisms associated with sudden roof collapse in longwall faces. Basarir et al.^14^ applied the global–local modeling approach to analyze the stability of the longwall roadways, which is applicable for roadways experiencing large convergence. Kang et al.^15^ indicated the front abutment stresses ahead of the coalface based on the load transfer mechanics. Esterhuizen et al.^16^ developed a numerical model to analyze tailgate stability at two longwall mines based on the field monitoring data. Darvishi et al.^17^ also developed a numerical method to investigate the effect of simultaneous extraction of two longwall panels on the stability of the maingate roadway, and indicated a good agreement with instrumentation data. Rajwa et al.^18^ modeled different variants of support designs to investigate the interactions between powered supports and rock mass during longwall mining.

In real-world situations, there are various types of experiments; just like support designing for underground coal mining, which seems to be a complicated problem. In this regard, the Design Of Experiments (DOEs) is a practical approach to describe the variation of information under hypothesized conditions. When the ranges of treatments are continuous, the relationship between the dependent and independents variables may be unknown. Therefore, Response Surface Methodology (RSM) aims at approximating the response function $y=f\left({x}_{1},{x}_{2},{x}_{3},\dots {x}_{n}\right)+e$. In other words, RSM searches an optimal response in a sequence of designed experiments to find the relationship between dependent and independent variables^19^.

RSM as an effective technique was employed in different fields of studies for modeling and optimizing processes related to the rock structures. Mollon et al.^20^ utilized a finite difference based RSM to probabilistically analyze the mechanized shallow tunnels in frictional and/or cohesive soils. Lü et al.^21^ employed RSM to investigate the reliability analysis of ground–support interaction in tunnels. Shamekhi and Tannant^22^ developed a methodology for probabilistic rock slope stability assessment using RSM and finite element numerical models. Park and Park^23^ employed RSM and numerical modeling to propose an inverse analysis method for the identification of the rock parameters around tunnels. Raina and Murthy^24^ predicted the flyrock distance in open pit blasting using a simplified RSM equation without compromising on the actual field applications. Abdallah and Verdel^25^ employed RSM together with numerical and experimental analyses to study the impact of mining subsidence on masonry buildings. Hamrouni et al.^26^ developed a RSM model to obtain the bending moment on lining and the surface settlement curve due to tunneling. Rastbood et al.^27^ developed a finite element based RSM to optimize the geometrical and mechanical characteristics of tunnel lining segments. Ozfirat et al.^28^ employed RSM to investigate the relations between independent variables of geomechanical and machine properties to predict the performance of roadheaders in an underground metal mine. Park and Park^29^ proposed a back analysis approach by combining RSM and finite difference methods to estimate the geotechnical features. And, Heidarzadeh et al.^30^ applied RSM along with numerical modeling to assess the effect of geometry on rock damage.

This research develops an RSM model based on the geomechanical parameters and numerical simulations to optimize support systems in longwall tailgates. In addition, the most important parameters affecting roof strata stability are analyzed and determined to predict unstable zones around the tailgate T-junction in advance of mining.

## Problem statement

**Figure 1:**
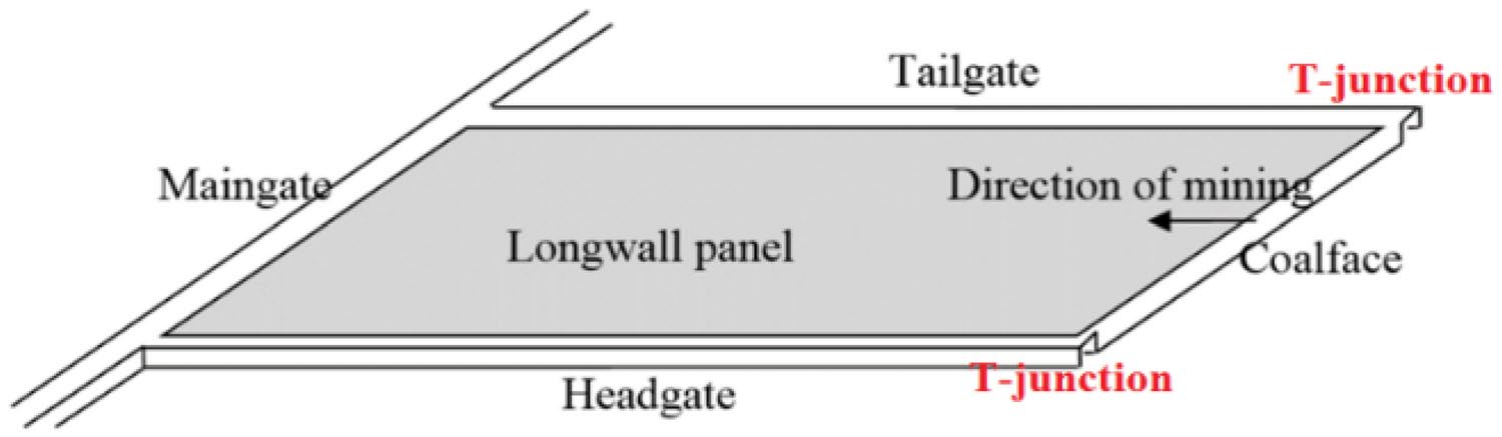
Schematic view of coalface, roadways, and T-junctions in a longwall panel.

Coal or any other layered deposits could be extracted by the longwall mining method through dividing a reserve in some large rectangular blocks, known as a panel. Before exploitation, each panel is developed by driving a set of tunnels or roadways on either side of the panel off the main access roadways. The two tunnels on each side of the panel are necessary to develop the coalface. Each tunnel serves a unique function; headgate for the transportation of material and personnel, and tailgate for escape way and ventilating the return air. The working face is created through a slot by joining these two roadways, and the coalface is supported by hydraulic roof supports to provide a safe working space underground (Fig. 1).

During longwall mining, the immediate roof, which is two to eight times the thickness of the coal seam, extensively fractures, and falls into the mined-out area. After face advancement, a goaf is formed as the immediate roof is allowed to cave behind the support systems.

In practice, some adjacent longwall panels are designed to exploit in turn. Therefore, a headgate in the previous panel should play the role of a tailgate in the next one. This issue leads to a high-stress distribution around the tailgate roadway due to the superposition of abutment stresses resulted from the two adjacent panels. The headgate is always located on the solid coal side of the reserve, where the next panel is located; so that it is not influenced by the side abutment stresses generated by the previous mined-out panel. While the tailgate roadway within a multiple extraction panel endures a wide range of loading conditions over its services^9^. Abutment stresses on the tailgate may cumulate from induced stresses due to exploitation in current and previous panels, and also from coalface passing and goaf caving in the current panel. This issue causes a high-stress concentration in the vicinity of the T-junction in the tailgate roadway.

**Figure 2:**
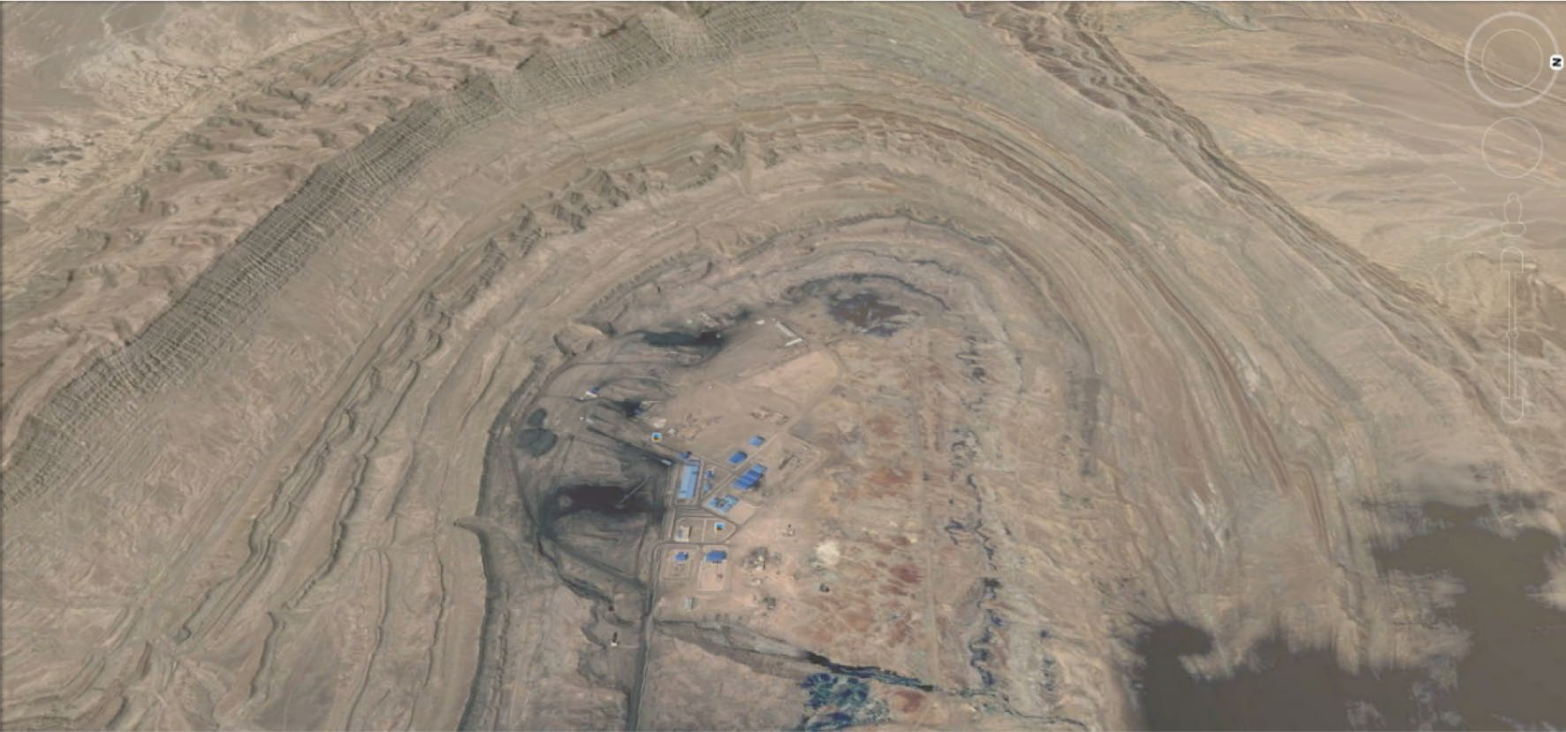
Location and structural geology of the Tabas mine region, taken from GoogleEarth^32^.

The problem of tailgate instability is a common adversity in the Tabas longwall mine, Parvadeh coalfield, South Khorasan province, Iran. As shown in Fig. 2, the Parvadeh coalfield is in a coal basin bounded by two major north–south trending fault systems^31^. The mine is developed in the southern limb on the south side of the Rostam fault, where the asymmetric Parvadeh anticline lies.

**Figure 3:**
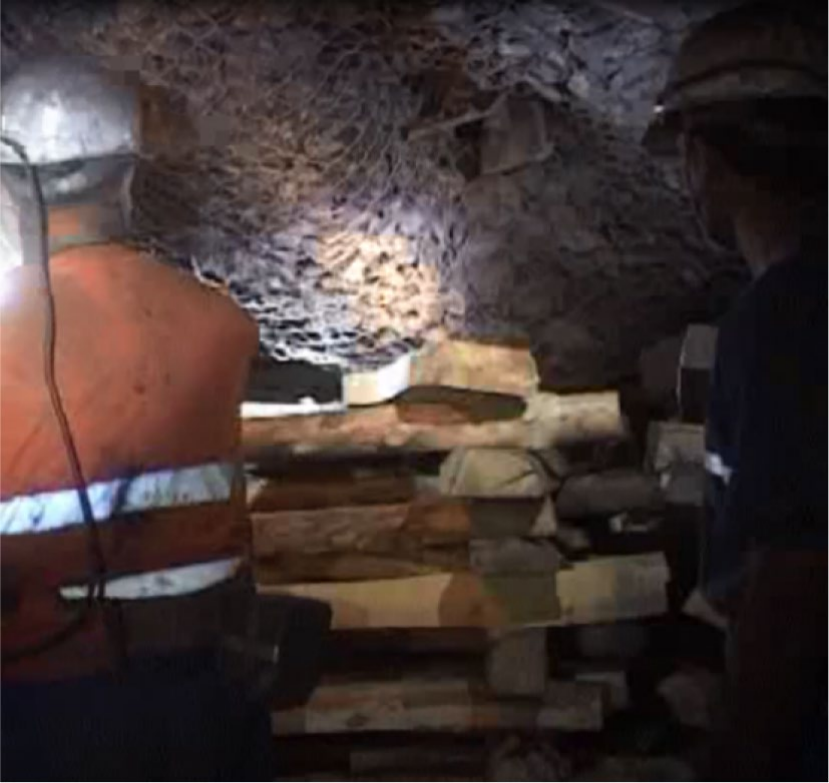
Severe roof displacement in the tailgate roadway at Tabas mine.

Due to the severe deformations of the rock strata along with tight folding and numerous faults in the zones adjacent to the Rostam fault, major instabilities frequently occur in the vicinity of the northern boundary of the mining area. Therefore, many mine downtimes are reported because of destructive failures taken place as results of the unstable support systems in the tailgate roadway and its T-junction (Fig. 3).

Field observations show that the coal-bearing strata in the Tabas basin consists mainly of sediments of the Upper Triassic-Middle Jurassic era namely the Nayband formation and Qadir member, about one kilometer in thickness. The rocks are mostly mudstone, siltstone, sandstone, and limestone sequences. The main coal seams are within a 50 m section of the central strata. The main seam thickness varies from 2.2 to 1.5 m, and the gradients vary from 1 in 2 to 1 in 9^31^.

This research is conducted to optimize the support systems at the tailgate roadway and to indicate the most effective parameters on the T-junction instabilities. The problem of unstable T-junctions especially in the vicinity of the intersection of coalface and tailgate roadway, as a problematic disaster in Tabas longwall mine, is focused to be analyzed using the finite-difference based RSM.

## Theory

### Response surface methodology

RSM is a commonly used mathematical method for optimizing the problems, which are affected by some variables in responses of experiments^33^. The primary objective of RSM is optimization, finding the best set of factor levels to attain some goals in a multi-purpose strategy. In other words, RSM combines the DOEs, optimization methods, and regression analyses to develop a suitable functional relationship between some independent variables and a response of interest ($y$) denoted by ${x}_{i}$ ($y=f\left({x}_{1},{x}_{2},{x}_{3},\dots {x}_{n}\right)+e)$^34^. The independent variables are also called factors, input variables or process variables.

RSM was first presented in the 1950s as a sequential procedure applied to chemical processes design by Box and Wilson^35^. Afterward, the RSM capability in solving complex problems causes to the reduction of experimental runs and statistically acceptable results, which lead to efficaciously extensive applications of RSM in different fields of engineering. In order to introduce the formulation of the RSM, it can mathematically be written as:

1 $$\mathrm{max}f\left(x\right)\equiv \mathrm{E}\left(Y\left(x\right)\right)$$

where $Y$ is a set of random variables having two parameters; a mean as an unknown function of the $q$-dimensional factor $x$, and a variance as an unknown constant value denoted by ${\sigma }^{2}$.

RSM matches a sequence of local regression models, which are fitted to experimental data based on the DOE. The most common models are linear, two-factor interaction (2FI), quadratic, and cubic, which will respectively be written as:

2 $$Y\left(x\right)={\beta }_{0}+\sum_{i=1}^{q}{\beta }_{i}{x}_{i}+\varepsilon \quad {\text{Linear}}$$

3 $$Y\left(x\right)={\beta }_{0}+\sum_{i=1}^{q}{\beta }_{i}{x}_{i}+\sum_{i<j=2}^{q}{\beta }_{ij}{x}_{i}{x}_{j}+\varepsilon \quad {\text{2FI}}$$

4 $$Y\left(x\right)={\beta }_{0}+\sum_{i=1}^{q}{\beta }_{i}{x}_{i}+\sum \sum_{i<j=2}^{q}{\beta }_{ij}{x}_{i}{x}_{j}+\varepsilon \quad {\text{Quadratic}}$$

5 $$Y\left(x\right)={\beta }_{0}+\sum_{i=1}^{q}{\beta }_{i}{x}_{i}+\sum \sum_{i<j=2}^{q}{\beta }_{ij}{x}_{i}{x}_{j}+\sum \sum \sum_{i<j<k=3}^{q}{\beta }_{ijk}{x}_{i}{x}_{j}{x}_{k}+\varepsilon \quad {\text{Cubic}}$$

where ${\beta }_{0}$ is the constant term, $q$ is the number of variables, ${\beta }_{i}$ are the linear coefficients, ${\beta }_{ij}$ and ${\beta }_{ijk}$ represent the interaction coefficients, ${x}_{i}$, ${x}_{j}$ and ${x}_{k}$ are the process variables, and $\varepsilon$ is the residual. The coefficients are obtained based on the least-squares method in such a way that the sum of the squares of the errors, $\varepsilon$, is minimized. For $n$ runs, $\varepsilon$ is a normally distributed random $n$-vector with mean the zero vector and covariance matrix ${\sum }_{\varepsilon }={\sigma }^{2}I$.

In order to find a suitable approximation between the independent and dependent variables, the second-order models are commonly used in RSM due to some advantages such as flexibility, variety of functional forms, and using the least-squares method. In order to build the second-order models, the Central Composite Design (CCD) was presented by Box and Wilson^35^ as a well-known experimental design.

### Central composite design

**Figure 4:**
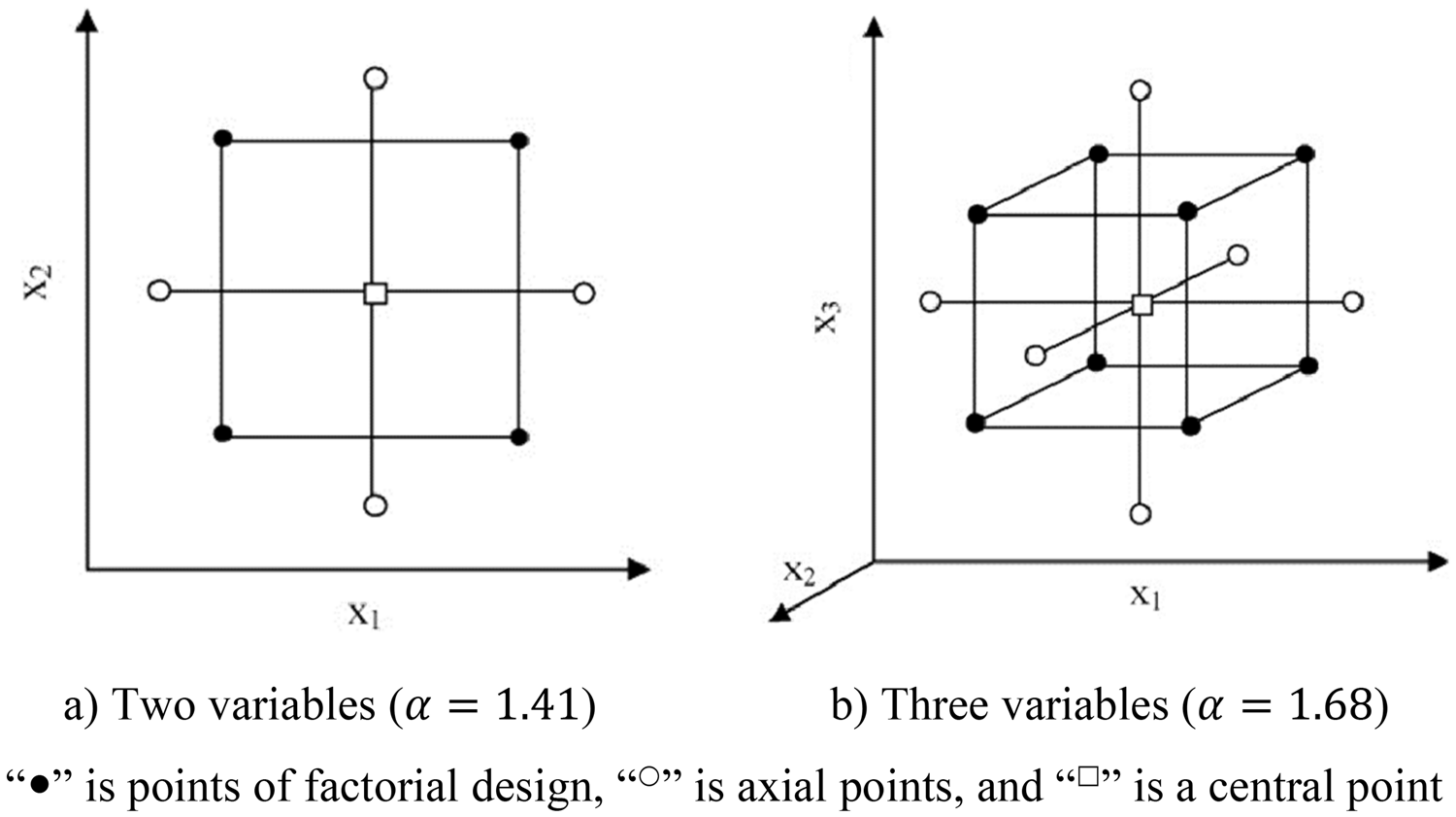
Schematic view of CCD for optimization of two and three variables.

CCD as a five-level RSM design, is a perfect solution for fitting a second-order model to optimize the response variable of interest by selecting corner, axial, and center points^35^. Although there are some varieties of CCD, the rotatable CCD is selected in our study as the experimental design method, which maintains the rotatability of the variation and assists in retaining the accuracy of model fitting. In addition, rotatable designs lead to equal prediction variance at all points in regression models, given a fixed distance from the center of the design (Fig. 4). The design procedure in CCD consists of three parts: (I) a fractional factorial design; (II) experimental points at the distances $\pm \alpha$ from their centers; and (III) a central point. Therefore, the whole factors in CCD are coded in five levels of $(-\alpha , -1, 0, +1, +\alpha )$ as presented in Table 1.

**Table 1:** Relationship between coded and actual values of a variable.

Quality	Coded value	Actual value
Lowest	$-\alpha$	${x}_{min}$
Low	$-1$	$\frac{{x}_{max}+{x}_{min}}{2}-\frac{{x}_{max}-{x}_{min}}{2\times {2}^{q/4}}$
Centre	$0$	$\frac{{x}_{max}+{x}_{min}}{2}$
High	$+1$	$\frac{{x}_{max}+{x}_{min}}{2}+\frac{{x}_{max}-{x}_{min}}{2\times {2}^{q/4}}$
Highest	$+\alpha$	${x}_{max}$

Where ${x}_{min}$ and ${x}_{max}$ are respectively the minimum and maximum values of $x$, and $q$ is the number of variables.

Transforming the input variables to coded variables, leads to being dimensionless with mean zero and the same standard deviation. Therefore, in order to define the desired ranges of input variables, each factor is coded to lie at $0$ for the center points, at $\pm 1$ for the factorial points, and at $\pm \alpha$ for the axial points. The axial points are rotatability selected to keep the variance for all points equidistant from the center.

### Finite difference method

The finite difference method (FDM) is one of the oldest numerical methods used for solving differential equations, given initial and boundary conditions. FDM was already known by Euler (1707–1783), and was later extended by Runge (1856–1927). The advent of FDM in numerical simulations began in the early 1950s and its progress was stimulated by the emergence of computers that provided a convenient framework for coping with complex engineering problems.

In the FDM, derivatives in several governing equations are directly substituted by an algebraic expression such as stress or displacement at discrete points in space, which are undefined within elements. The theory and background of FDM is summarized in the user manual of FLAC (Fast Lagrangian Analysis of Continua) software^36^. In this research, the numerical modeling is executed by FLAC software, which is an explicit finite difference program developed for solving complex mechanical problems in mining engineering and geotechnics.

## Results and discussion

**Figure 5:**
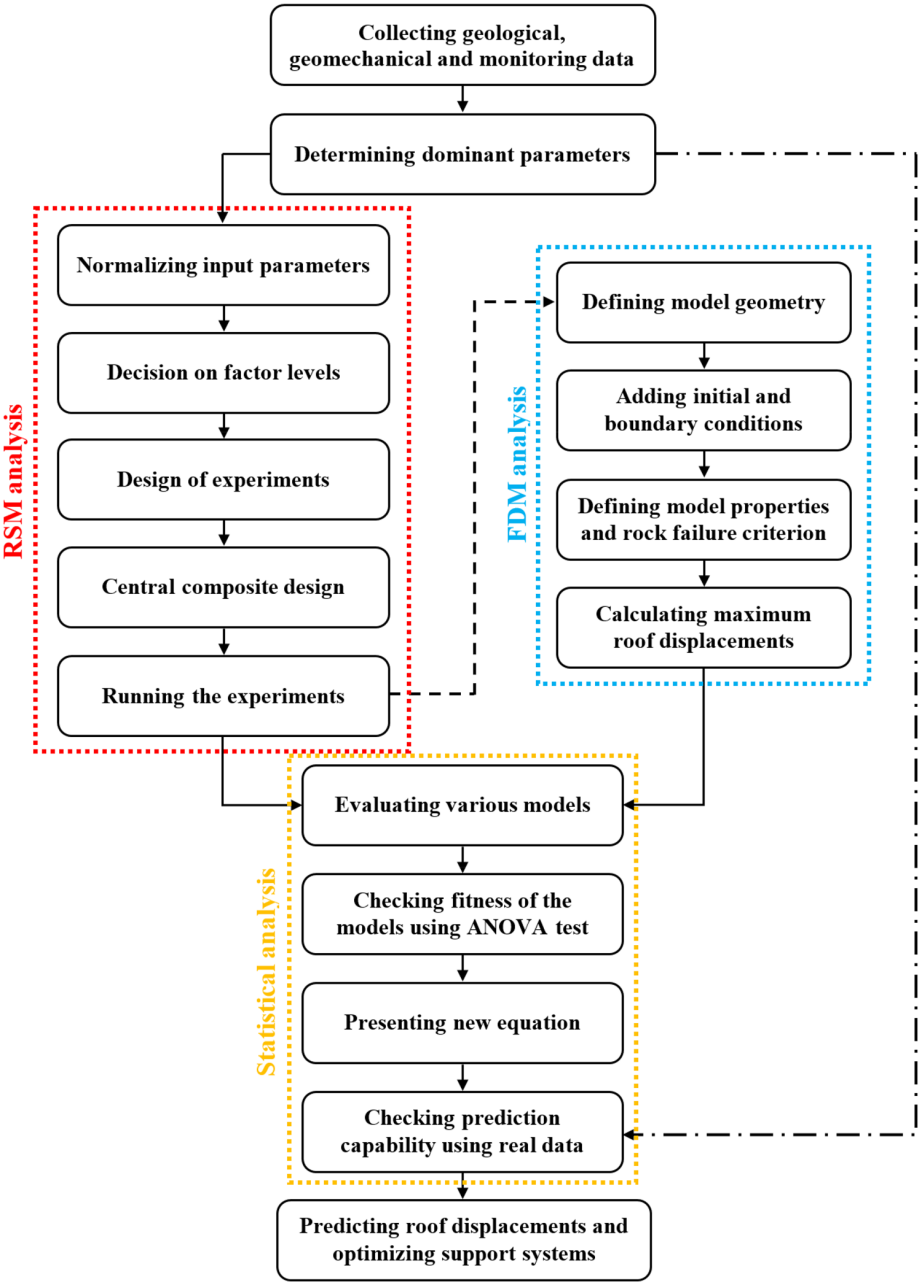
Steps of the proposed model for optimization of support systems in longwall tailgates.

As mentioned, RSM searches the relationships between some independent variables and one or more response variables in a sequence of designed experiments to attain an optimal response. RSM is employed in this research together with FDM and statistical analyses to examine the roof strata stability in longwall tailgate, and consequently to indicate the more sensitive parameters affecting the support systems instabilities in the tailgate roadway. For this purpose, some geomechanical parameters affecting roof displacements in the tailgate roadway of panel E2 at the Tabas longwall coal mine were selected. After screening and determining dominant parameters, the ranges of maximum and minimum changes of the influencing factors on maximum roof displacements (${d}_{max}$) are calculated. A DOE is then developed through considering a CCD on input variables which causes to 149 experiments. Thereafter, the ${d}_{max}$ values for each experiment are estimated for the whole possible conditions through some FDM numerical simulations in FLAC software. Finally, investigating various linear, 2FI, quadratic, and cubic models, a quadratic equation is developed to predict the ${d}_{max}$ in the tailgate roadway through statistical analyses. In addition, the dominant factors affecting the response variable (${d}_{max}$), and also the interaction among input variables are determined by statistical techniques. The developed procedure is summarized in Fig. 5.

### Determining dominant parameters

The first requirement for RSM is implementing a DOE framework or factorial design to achieve a reliable and suitable measure for the response of interest. For this purpose, a sequential procedure is followed to investigate more important factors influencing the response, and removing the unimportant ones. A fractional factorial design based on the CCD is employed in our research to design the experiments. The fractional factorial design is used to diminish the candidate variables, and consequently reduce the number of simulations. This issue leads to more proficient of the subsequent experiments by determining the dominant factors affecting the response variable. Since the number of design points required to fit a regression model enhances with the number of factors, screening out unimportant parameters can extensively reduce experimental efforts and computational complexity of the model.

After implementing a fractional factorial design, the important independent variables to optimize the support systems in panel E2 at Tabas longwall mine are obtained as density ($\rho$), uniaxial compressive strength ($UCS$), cohesion ($C$), angle of internal friction ($\phi$), slake durability index (${I}_{d2}$), Young’s modulus ($E$), shear strength ($\tau$), tensile strength (${\sigma }_{t}$), and rock mass rating ($RMR$). The input variables are collected from the rock mechanics information resulted from the field and laboratory studies based on the boreholes and geological reports which are gathered during mine development in the 1200 m long tailgate roadway.

### Normalizing input parameters

The input parameters are generally called the natural variables, since they are measured from the field or in the rock mechanics laboratory, and also they are expressed in the natural units of measurement. In RSM analysis, the natural variables are to be converted to the coded variables for dimensionless based on the equations presented in Table 1. The input parameters and the coded/actual values used in our research are summarized in Table 2.

**Table 2:** Coded and actual levels of input variables.

Symbol	Unit	$-\alpha$	$-1$	$0$	$+1$	$+\alpha$
$\rho$	g/cm^3^	1.600	1.887	2.175	2.462	2.750
$UCS$	MPa	3.500	53.200	102.900	152.600	202.300
$E$	GPa	0.340	2.992	5.645	8.297	10.950
$C$	MPa	0.010	2.557	5.105	7.652	10.200
$\phi$	deg	17.800	23.125	28.450	33.775	39.100
$\tau$	MPa	2.000	12.400	22.800	33.200	43.600
${\sigma }_{t}$	MPa	0.200	4.850	9.500	14.150	18.800
${I}_{d2}$	%	35.000	50.750	66.500	82.250	98.000
$RMR$	–	15.000	34.000	53.000	72.000	91.000

### Design of experiments

When coded and actual levels of natural variables are determined, the ranges of maximum and minimum changes for influencing parameters at this mine are calculated. A DOE is then developed through considering a CCD on independent variables, which results in 149 experiments. The rotatable CCD is implemented to design 149 runs with an appropriate combination for the factors $\rho$, $UCS$, $E$, $C$, $\phi$, $\tau$, ${\sigma }_{t}$, ${I}_{d2}$, and $RMR$. Finally, a five-level nine-variable CCD is adopted in our research to describe the response surfaces. The experiments are designed with Design Expert (DX7) software to choose and fit a suitable model to the experimental results.

### Numerical simulations

The 149 experiments resulted from the output of CCD, are introduced to FLAC software in order to calculate the ${d}_{max}$ in each experiment based on the FDM numerical analysis. Therefore, the maximum crown displacement at the roof of each modeled tailgate is recorded as the response of the design. Later, these results will be compared with the monitored roof displacements to investigate the validity of the model.

In numerical modeling, much attention is paid to follow the real conditions of the tailgate roadway. Therefore, the trapezoidal cross-section, inclined coal seam, and the arrangement of installed rockbolts are relatively simplified and modeled similar to the real conditions.

**Figure 6:**
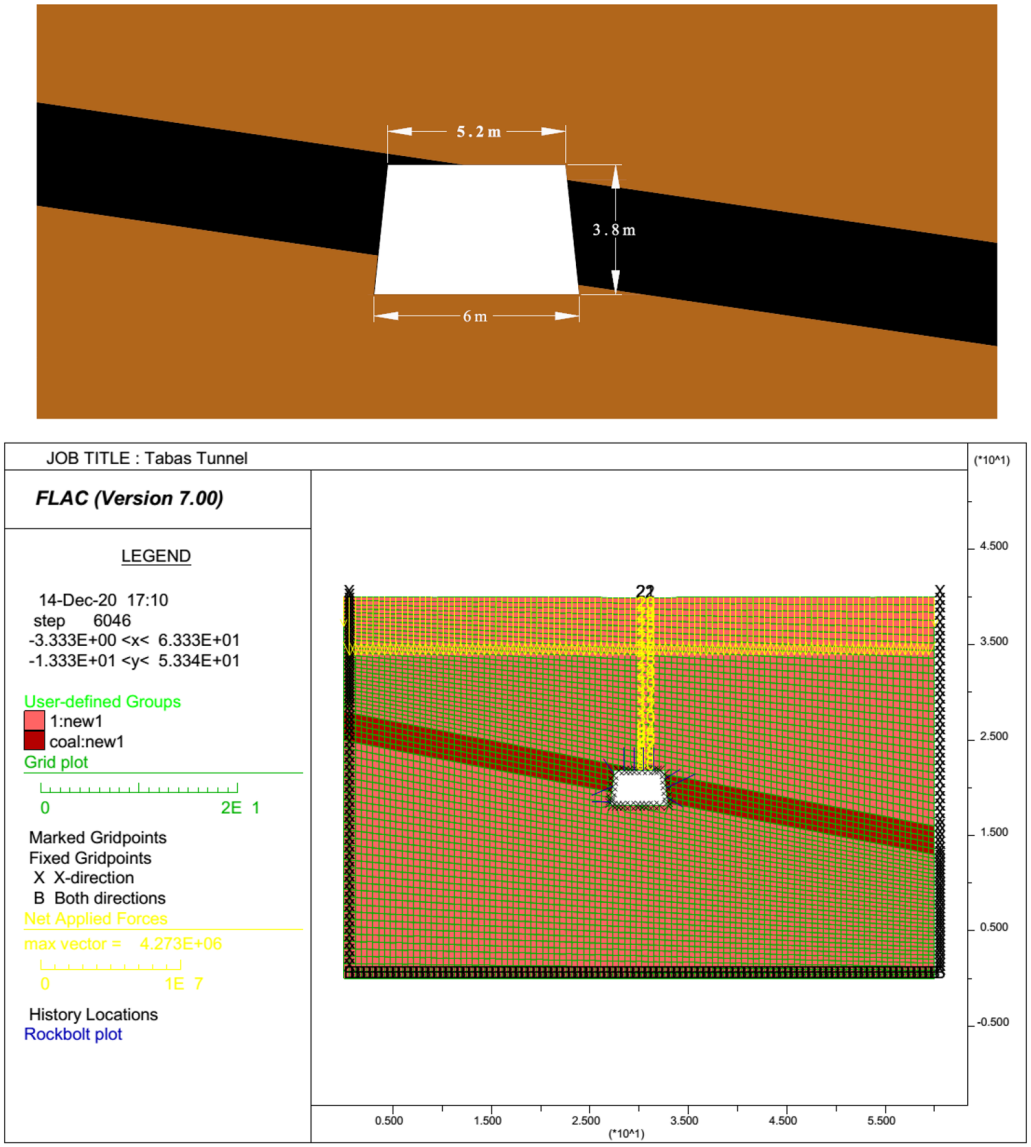
Geometry of the longwall tailgate modeled in FLAC software.

The geometry of a numerical model simulated in FLAC software is presented in Fig. 6. The model size was taken as 60 m in the x-direction, and 40 m in the y-direction. The model geometry was selected based on the Saint Venant principle, which neglects the effects of excavation out of a domain with 3 to 5 times the roadway width or radius. The roadway was set in the middle of the model, and a 27 m wide region at both sides of the model was preserved to eliminate the boundary effects. By defining this geometry, the stress and displacement distributions out of the model dimensions can be considered as being scarcely influenced by excavation.

The Mohr–Coulomb failure criterion was used as the constitutive model, and the initial and boundary conditions were simplified and added to each FDM model based on the real conditions. The geometrical and geomechanical properties, which were used in FDM modeling are the height of overburden, density of rock strata, Young’s modulus, Poisson’s ratio, cohesion, and angle of internal friction. A vertical stress in the range of 6.8 to 7.4 MPa, equivalent to the overburden weight, was applied to the upper boundary of the models. The horizontal displacement of the lateral boundaries, and the vertical and horizontal displacements of the bottom boundary were fixed.

**Figure 7:**
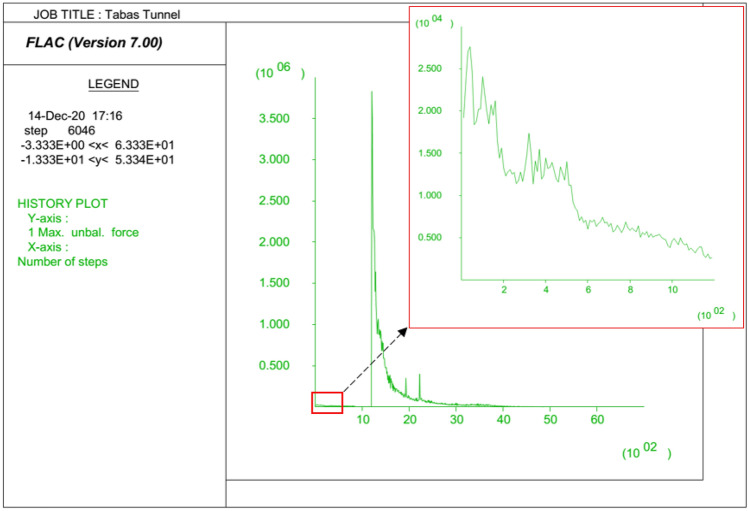
History of maximum unbalanced force for the selected section.

There are several ways to make sure that equilibrium has been reached in numerical modeling. In our research, a quick check was made by plotting the changes in maximum unbalanced force during running the FDM models. A plot of maximum unbalanced force versus timestep for the 5th section is presented in Fig. 7, in which the maximum unbalanced force before excavation of the roadway has been zoomed. As seen, the maximum unbalanced force in any stage of modeling is approaching zero, which indicates that an equilibrium state has been reached.

**Figure 8:**
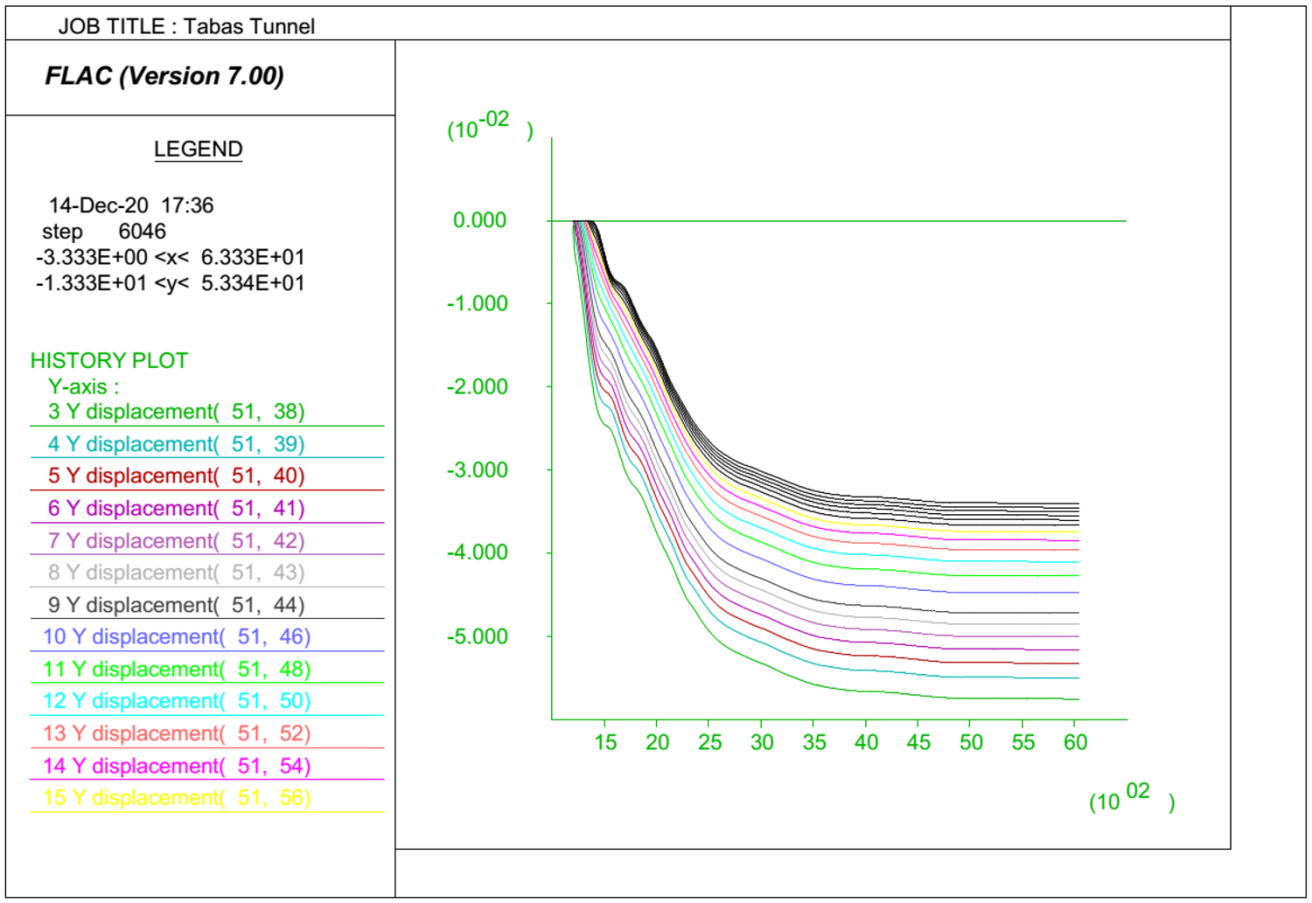
y-Displacement histories in specified gridpoints versus timestep for the selected section.

**Figure 9:**
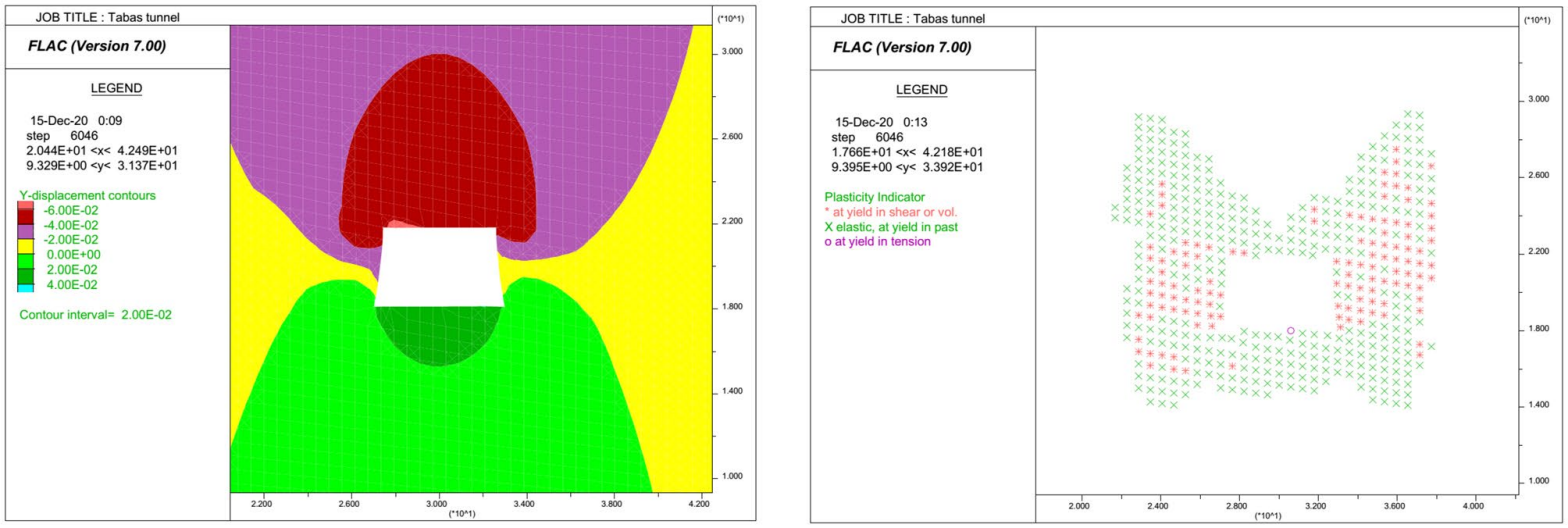
Plots of the y-displacement contours and plasticity indicator in the selected section.

In order to monitor the roof displacements during running the FDM model, the history of y-displacements at specified gridpoints are plotted versus timestep. The result of the FDM analysis for the selected section is presented in Fig. 8, showing a ${d}_{max}$ of 60 mm. The y-displacement contours and plasticity indicator resulted from analysis in this section are also presented in Fig. 9. Running each FDM model, the changes in the corresponding roof strata displacements are recorded. The results of the numerical analyses were verified based on the roof displacement recorded by dual-height telltales that were installed in the tailgate roadway. The procedure of numerical modeling was repeated in turn for the whole of 149 experiments.

### Statistical analysis

Now a comprehensive data are gathered based on the natural geological and rock mechanical information, as well as the numerical modeling in such a way that could cover all the possible conditions of instabilities that occurred at Tabas longwall mine. Since the experiments are designed based on the ranges of maximum and minimum changes of natural parameters, the whole scenarios governing the tailgate instability at the mine will be considered. The part of CCD with the coded/actual values (input variables) and the results of numerical simulations (${d}_{max}$) are given in Table 3.

**Table 3:** Part of CCD consisting of nine experimental factors and numerical results.

Run no.	Input variables									Response
	$UCS$	${\sigma }_{t}$	$C$	$\phi$	$E$	$\tau$	$\rho$	${I}_{d2}$	$RMR$	${d}_{max}$
1	152.60	4.85	2.56	23.13	2.99	12.40	1.89	82.25	72.00	52
2	152.60	4.85	7.65	33.78	8.30	12.40	2.46	50.75	72.00	68
3	152.60	14.15	2.56	23.13	8.30	33.20	2.46	82.25	34.00	41
–	–	–	–	–	–	–	–	–	–	–
–	–	–	–	–	–	–	–	–	–	–
149	102.90	9.50	0.01	28.45	5.64	22.80	2.17	66.50	53.00	97

In order to construct the linear, 2FI, quadratic, and cubic response surfaces of the ${d}_{max}$, the output of the RSM models are statistically analyzed based on the fractional factorial design. The RSM establishes a mathematical relation between the input geomechanical parameters and the ${d}_{max}$, which may be applied as a predictive model for estimating the stability behavior of the longwall tailgate without further complicated and time-consuming numerical simulations.

**Figure 10:**
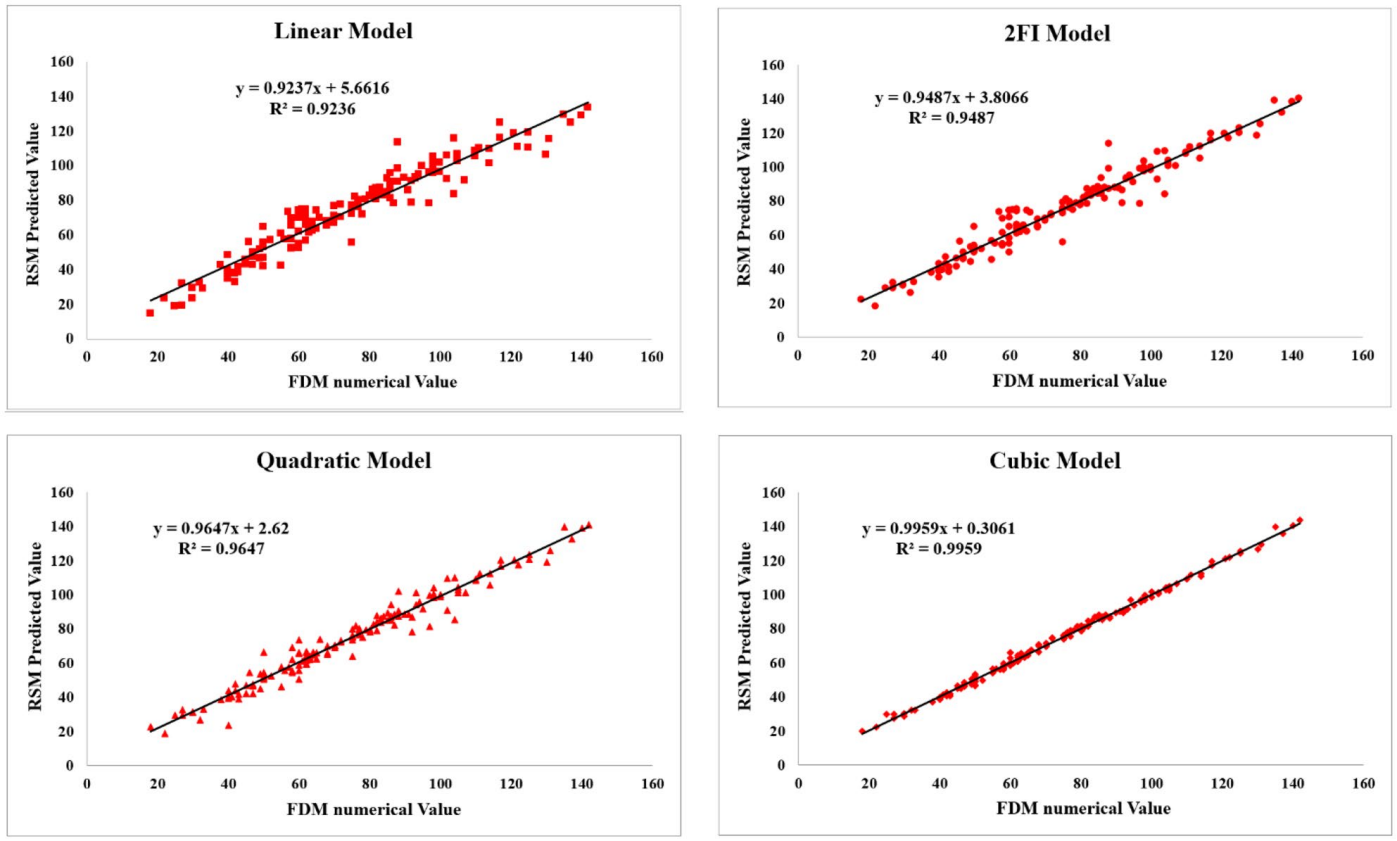
Comparison of the RSM and FDM values of ${d}_{max}$ in linear, 2FI, quadratic and cubic models.

The results of the coefficient of determination (${R}^{2}$) for the linear, 2FI, quadratic and cubic models are respectively determined as 0.9236, 0.9487, 0.9647, and 0.9959, and are illustrated in the form of the FDM numerical values versus RSM predicted ones in Fig. 10.

### Proposed model

In order to optimize support systems in the tailgate roadway, the key variables affecting roof displacements are at first identified. For this purpose, a set of adjustments are implemented on the resulted equations of the linear, 2FI, quadratic, and cubic models to move the process toward the optimum conditions. At the next step, the interactions between more important independent variables are recognized. In order to investigate the effect of multiple input parameters, and their interactions on the ${d}_{max}$, the analysis of variance (ANOVA) is also employed. Finally, the most effective parameters on ${d}_{max}$, and the pairwise interaction effects between effective parameters are investigated and determined.

Analyzing different scenarios, a modified quadratic equation was obtained to predict the ${d}_{max}$ by introducing the geomechanical parameters $\rho$, $UCS$, $E$, $C$, $\phi$, $\tau$, ${\sigma }_{t}$, ${I}_{d2}$, and $RMR$. The selected independent variables are of importance, and usually are to be available in designing almost all rock structures. Therefore, the developed model can be employed in practice by using archive geomechanical information to gain a better understanding of the roadway stability ahead of time. After running DOEs, the final proposed equation in terms of coded factors is obtained as:

6 $$ \begin{aligned}{d}_{max}&={\left(\vphantom{\left.+0.047 C \tau  +0.24 {RMR}^{2}+0.15 {C}^{2}-0.12 {\rho }^{2}+0.11 {\tau }^{2}+0.099 {{\sigma }_{t}}^{2}\right)}8.04-1.17 {I}_{d2}-0.72 E-0.56 UCS-0.55 RMR-0.26 \tau -0.14 {\sigma }_{t}-0.13 C -0.029 \phi +0.027 \rho \right.}\\&\quad {\left.-0.13 \phi E+0.079 \tau RMR-0.073 UCS RMR -0.065 UCS {I}_{d2}+0.057 \tau {I}_{d2}-0.051 C {I}_{d2}-0.049 E {I}_{d2}\right.}\\&\quad {\left.+0.047 C \tau  +0.24 {RMR}^{2}+0.15 {C}^{2}-0.12 {\rho }^{2}+0.11 {\tau }^{2}+0.099 {{\sigma }_{t}}^{2}\right)}^{2}\end{aligned} $$

The final proposed equation in terms of actual parameters is also obtained as:

7 $$ \begin{aligned}{d}_{max}&={\left(\vphantom{\left.+0.0227 {C}^{2}-1.4993 {\rho }^{2}+0.0010 {\tau }^{2}+0.0046 {{\sigma }_{t}}^{2}\right)}12.3920-0.0608 {I}_{d2}+0.0755 E-0.0017 UCS-0.0992 RMR-0.1239 \tau  -0.1181 {\sigma }_{t}\right.}\\&\quad{\left.-0.2399 C+0.0484 \phi +6.6150 \rho -0.0095 \phi E+0.0004 \tau RMR-0.00008 UCS RMR\right.}\\&\quad{\left.-0.00008 UCS {I}_{d2}+0.0004 \tau {I}_{d2} -0.0013 C {I}_{d2}-0.0012 E {I}_{d2}+0.0018 C \tau +0.0007 {RMR}^{2} \right.}\\&\quad{\left.+0.0227 {C}^{2}-1.4993 {\rho }^{2}+0.0010 {\tau }^{2}+0.0046 {{\sigma }_{t}}^{2}\right)}^{2}\end{aligned} $$

**Figure 11:**
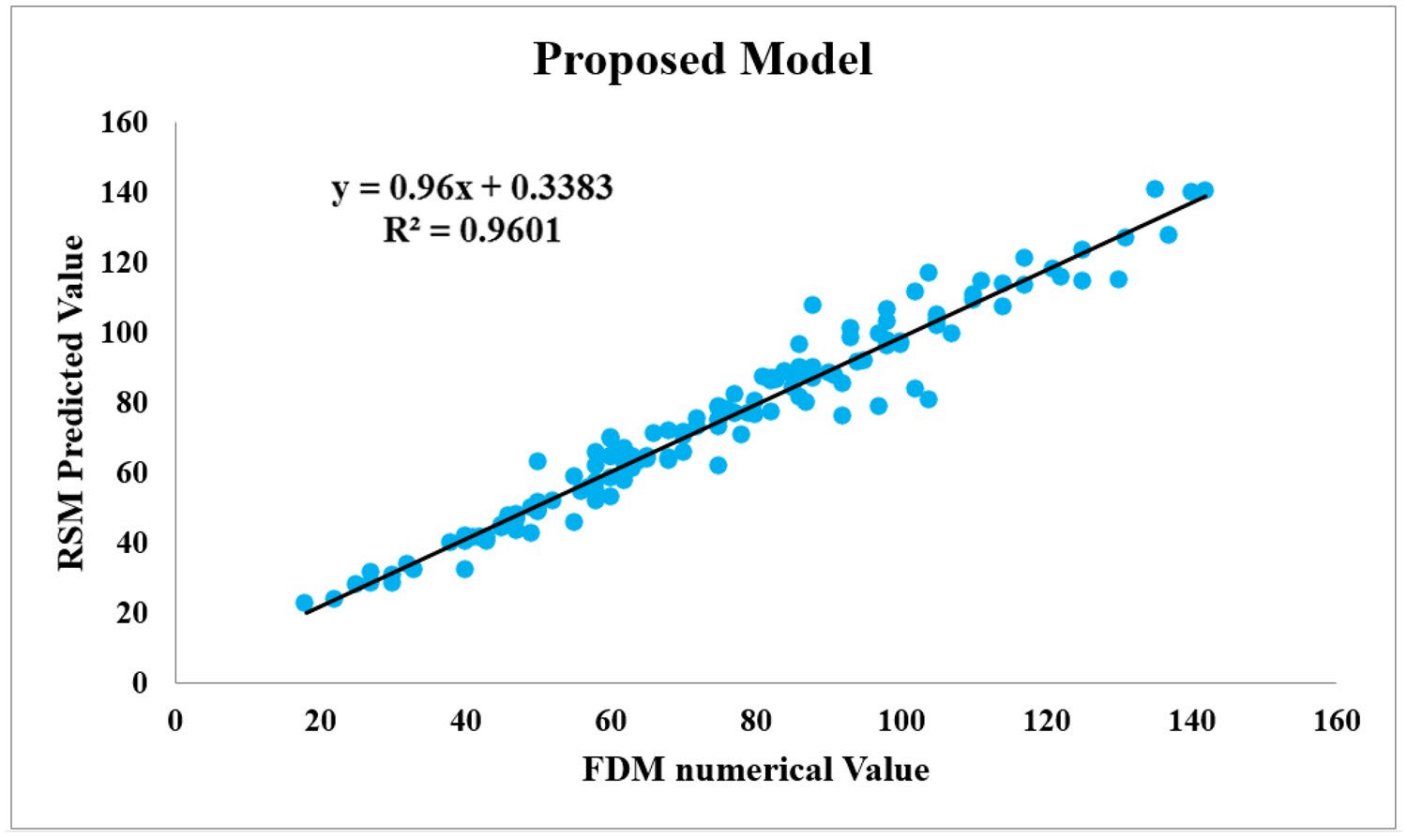
Comparison of the RSM and FDM values of ${d}_{max}$ in the proposed model.

Figure 11 illustrates the results of the RSM predicted ${d}_{max}$ versus the FDM numerical values for the proposed model. As seen, ${R}^{2}$ is obtained 0.9601, which indicates a high goodness of fit. Due to the high level of accuracy, the proposed model is relatively reliable and may be useful in predicting roof displacements at tailgate roadway of panel E2 ahead of time in Tabas longwall mine. It is also applicable to the other similar longwall mines after validation and verification.

In Table 4, the model-fitting procedure is presented based on the regression output from DX7 software. Evaluation criteria for four models of linear, 2FI, quadratic, and cubic are presented in this table together with the evaluation criteria for our proposed model. The significance of each model is assessed based on the MSE, F-value, *p* value, adequate precision, and PRESS tests, and also the coefficient of determination (${R}^{2}$), the adjusted coefficient of determination (${R}_{adj}^{2}$), and the predicted coefficient of determination (${R}_{pre}^{2}$).

**Table 4:** Evaluation criteria for investigated and proposed regression models.

Response surface	df	MSE	F-value	*p* value	Adeq precision	PRESS	${R}^{2}$	${R}_{adj}^{2}$	${R}_{pre}^{2}$
Linear model	9	11,156.44	186.80	< 0.0001	59.292	9279.57	0.9236	0.9187	0.9146
2FI model	45	2291.73	42.29	< 0.0001	29.896	8068.99	0.9487	0.9262	0.9212
Quadratic model	54	1942.00	47.52	< 0.0001	31.483	13,121.37	0.9647	0.9444	0.8793
Cubic model	118	917.48	61.53	< 0.0001	35.932	87,370.04	0.9959	0.9797	0.1963
Proposed model	22	16.63	137.95	< 0.0001	52.011	30.01	0.9601	0.9532	0.9258

The ${R}^{2}$, ${R}_{adj}^{2}$, and ${R}_{pre}^{2}$ can be calculated using the following equations. ^37^:

8 $${R}^{2}=\frac{{SS}_{R}}{{SS}_{T}}=1-\frac{{SS}_{E}}{{SS}_{T}}$$

9 $${R}_{adj}^{2}=1-\frac{\frac{{SS}_{E}}{\left(n-p\right)}}{\frac{{SS}_{T}}{\left(n-1\right)}}$$

10 $${R}_{pre}^{2}=1-\frac{PRESS}{{SS}_{T}}$$

where, ${SS}_{T}$, ${SS}_{E}$, and ${SS}_{R}$ are respectively the total sum of squares, the regression sum of squares, and the residual sum of squares. The $n$ and $p$ are also the sample size and the number of independent variables. The prediction capabilities of the investigated models are evaluated using the ${R}_{pre}^{2}$, which is obtained based on the Predicted Residual Error Sum of Squares (PRESS) value^38^. The PRESS statistic is computed in the leave-one-out cross validation process or jackknife technique, by adding the square of the residuals for the case that is left out^39,40^. The idea in the leave-one-out cross validation is to fit the model without the ${i}^{th}$ observation ${x}_{i}$, and use this fitted model to predict the response ${\widehat{y}}_{(i)}$ at ${x}_{i}$. In other words, the regression model is fitted to the remaining $n-1$ observations to predict the withheld observation ${y}_{i}$, which is denoted by ${\widehat{y}}_{(i)}$. This procedure will be repeated for all $n$ observations, producing a set of $n$ PRESS residuals,

and the PRESS statistic is computed as the sum of squares of the $n$ PRESS residuals^34,40^:

11 $$PRESS=\sum_{i=1}^{n}{\left({y}_{i}-{\widehat{y}}_{(i)}\right)}^{2}=\sum_{i=1}^{n}{e}_{(i)}^{2}$$

where ${y}_{i}$, ${\widehat{y}}_{(i)}$, and ${e}_{(i)}$ are respectively the observed value, the estimated value, and the prediction error. The mean sum of squares of error (MSE) is also obtained as:

12 $$MSE=\sum_{i=1}^{n}\frac{{\left({y}_{i}-{\widehat{y}}_{(i)}\right)}^{2}}{n-1}$$

A comparison of the results shows that the *p* value of the five models is less than 0.05 which indicates a well-developed procedure for the whole models. In addition, ${R}^{2}$ is more than 0.9 in all five models. Based on the ${R}_{adj}^{2}$ values, it appears that ${R}_{adj}^{2}$ for the cubic model is more than the proposed model value. However, the ${R}_{pre}^{2}$ proved that the cubic model has been entangled in the overfitting phenomenon. Hence, ${R}_{pre}^{2}$ in our proposed model in about 0.93, which shows a high level of accuracy for the prediction of ${d}_{max}$. Unlike the cubic and quadratic models, in our proposed model the value of 0.9258 for ${R}_{pre}^{2}$ is in reasonable agreement with a value of 0.9532 for ${R}_{adj}^{2}$. Examining the other statistical parameters such as MSE and PRESS, reveals the prediction proficiency of the proposed model in relation to the others. In addition, the adequate precision factor, which measures the signal to noise ratio is obtained 52.011 indicating an adequate and desirable ratio.

### Sensitivity analysis

In order to indicate the most sensitive parameters influencing the ${d}_{max}$ in the proposed model, the influences of the whole input parameters $\rho$, $UCS$, $E$, $C$, $\phi$, $\tau$, ${\sigma }_{t}$, ${I}_{d2}$, and $RMR$ on the ${d}_{max}$ are investigated through conducting a sensitivity analysis. For this purpose, the ANOVA is implemented on the results of the proposed model to evaluate the effect of each input parameter on the ${d}_{max}$, and also investigate their pairwise interactions. The results of ANOVA consisting of the sum of squares, degree of freedom (df), MSE, F-value, *p* value, and Variance Inflation Factor (VIF) are presented in Table 5.

**Table 5:** Results of ANOVA for the proposed model.

Source	Sum of squares	df	MSE	F-value	*p* value	VIF	Note
Model	365.91	22	16.63	137.95	< 0.0001		Significant
$\mathrm{UCS}$	42.54	1	42.54	352.82	< 0.0001	1.00	Significant
${\upsigma }_{\mathrm{t}}$	2.85	1	2.85	23.62	< 0.0001	1.00	Significant
$\mathrm{C}$	2.35	1	2.35	19.52	< 0.0001	1.00	Significant
$\upphi$	0.11	1	0.11	0.93	0.3368	1.00	
$\mathrm{E}$	71.28	1	71.28	591.21	< 0.0001	1.00	Significant
$\uptau$	9.50	1	9.50	78.80	< 0.0001	1.00	Significant
$\uprho$	0.097	1	0.097	0.81	0.3708	1.00	
${\mathrm{I}}_{\mathrm{d}2}$	186.43	1	186.43	1546.27	< 0.0001	1.00	Significant
$\mathrm{RMR}$	40.60	1	40.60	336.76	< 0.0001	1.00	Significant
$\mathrm{UCS}$ ${\mathrm{I}}_{\mathrm{d}2}$	0.54	1	0.54	4.45	0.0369	1.00	Significant
$\mathrm{UCS}$ $\mathrm{RMR}$	0.68	1	0.68	5.67	0.0188	1.00	Significant
$\mathrm{C}$ $\uptau$	0.28	1	0.28	2.34	0.1285	1.00	
$\mathrm{C}$ ${\mathrm{I}}_{\mathrm{d}2}$	0.33	1	0.33	2.73	0.1007	1.00	
$\upphi$ $\mathrm{E}$	2.31	1	2.31	19.20	< 0.0001	1.00	Significant
$\mathrm{E}$ ${\mathrm{I}}_{\mathrm{d}2}$	0.30	1	0.30	2.52	0.1150	1.00	
$\uptau$ ${\mathrm{I}}_{\mathrm{d}2}$	0.42	1	0.42	3.49	0.0642	1.00	
$\uptau$ $\mathrm{RMR}$	0.80	1	0.80	6.65	0.0111	1.00	Significant
${\upsigma }_{\mathrm{t}}^{2}$	0.34	1	0.34	2.81	0.0959	1.04	
${\mathrm{C}}^{2}$	0.75	1	0.75	6.24	0.0138	1.04	Significant
${\uptau }^{2}$	0.39	1	0.39	3.27	0.0730	1.04	
${\uprho }^{2}$	0.53	1	0.53	4.41	0.0378	1.04	Significant
${\mathrm{RMR}}^{2}$	1.93	1	1.93	16.00	0.0001	1.04	Significant
Residual	15.19	126	0.12				
Lack of fit	15.12	124	0.12	3.66	0.2388		Not significant
Pure error	0.067	2	0.033				
Cor total	381.10	148					

According to the results of ANOVA, the *p* value of the proposed model is under 0.05, which means a high accuracy for the prediction of ${d}_{max}$. The calculated F-value and *p* value for lack of fit are respectively 3.66 and 0.2388, which may imply that the proposed model is satisfactory, and the parameter of lack of fit is not significant in relation to the pure error. Also, based on the resulted F-value, the parameters ${I}_{d2}$, $E$, $UCS$, and $RMR$ are the key parameters influencing the ${d}_{max}$, while the parameters $\phi$ and $\rho$ do not have a significant effect on the response variable. Therefore, the parameters ${I}_{d2}$, $E$, $UCS$, and $RMR$ are the more sensitive factors, and the parameters $\phi$ and $\rho$ are the less ones. Amongst them, the ${I}_{d2}$ appears to be the most effective input variable on the ${d}_{max}$, meaning that by enhancement of the ${I}_{d2}$, the ${d}_{max}$ will sharply be decreased.

The other important parameter in the ANOVA table is VIF, which measures the upsurge of the variance in comparison with an orthogonal basis. The VIF of the ${i}^{th}$ variable is defined as:

13 $${VIF}_{i}=\frac{1}{1-{R}_{i}^{2}}$$

where ${R}_{i}^{2}$ is the ${R}^{2}$ value obtained by regressing the ${i}^{th}$ predictor on the remaining predictors.

In general, to develop a model with a high goodness of fit, the VIF of all input variables should be less than five to prevent dilemmas with the stability of the coefficients. When the VIF is obtained more than five, it is possible to present a severe multicollinearity. Therefore, the variables with the high values of VIF are not suitable and should be removed from the model. As shown in Table 5, the VIF is calculated for all independent variables, and the resulted VIF values are in the range of 1, showing a supreme situation for the proposed model.

**Figure 12:**
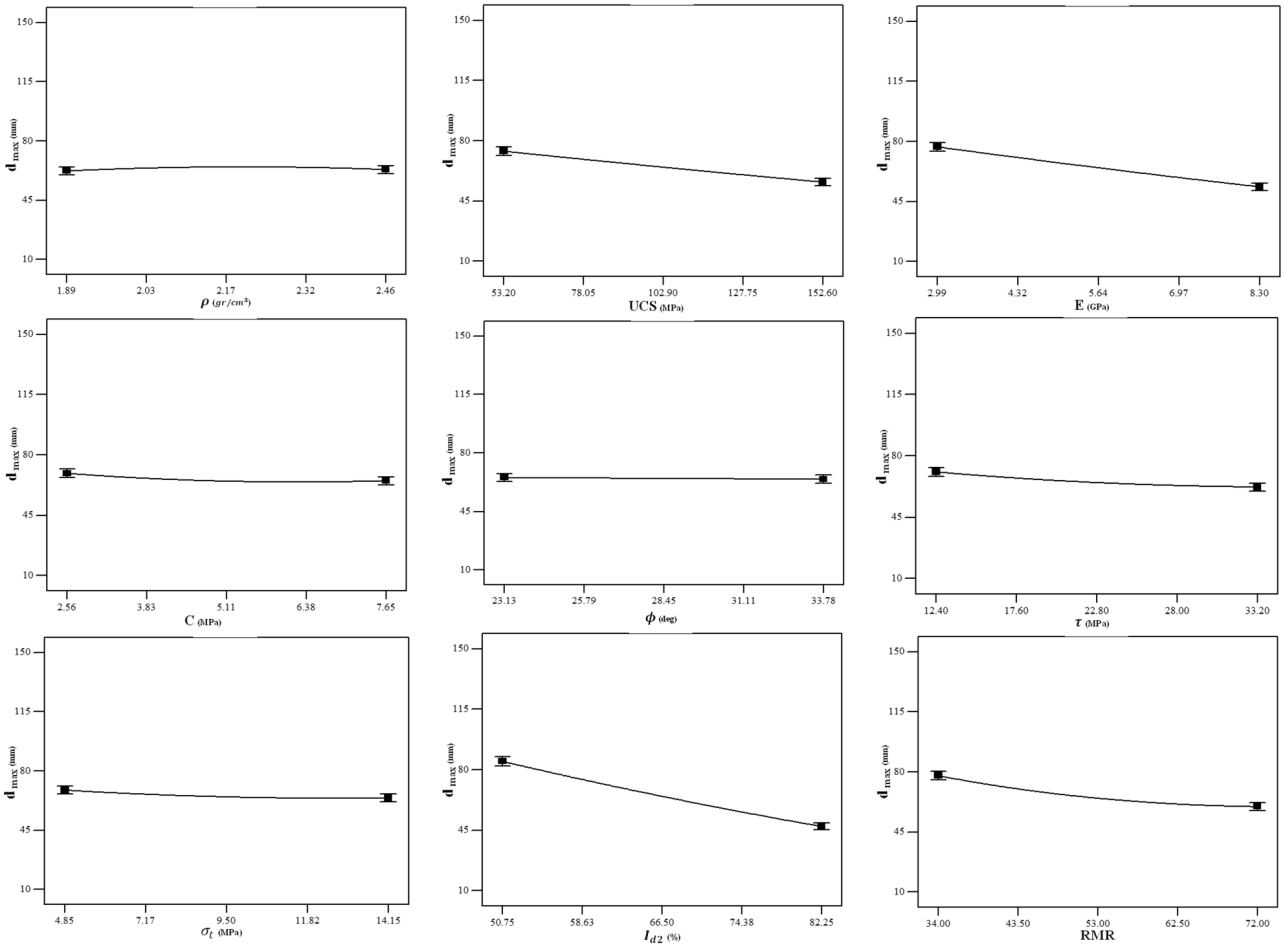
Influences of the input variables on the ${d}_{max}$.

The influences of the whole nine input parameters on the ${d}_{max}$ are depicted in Fig. 12 to provide a perception for comparison of the effect of each parameter on the ${d}_{max}$. As seen, the most sensitive parameters are ${I}_{d2}$, $E$, $UCS$, and $RMR$ meaning that the ${d}_{max}$ will be decreased when these parameters increase. Also, this figure reveals that the parameters $\phi$ and $\rho$ do not have a significant influence on the ${d}_{max}$.

**Figure 13:**
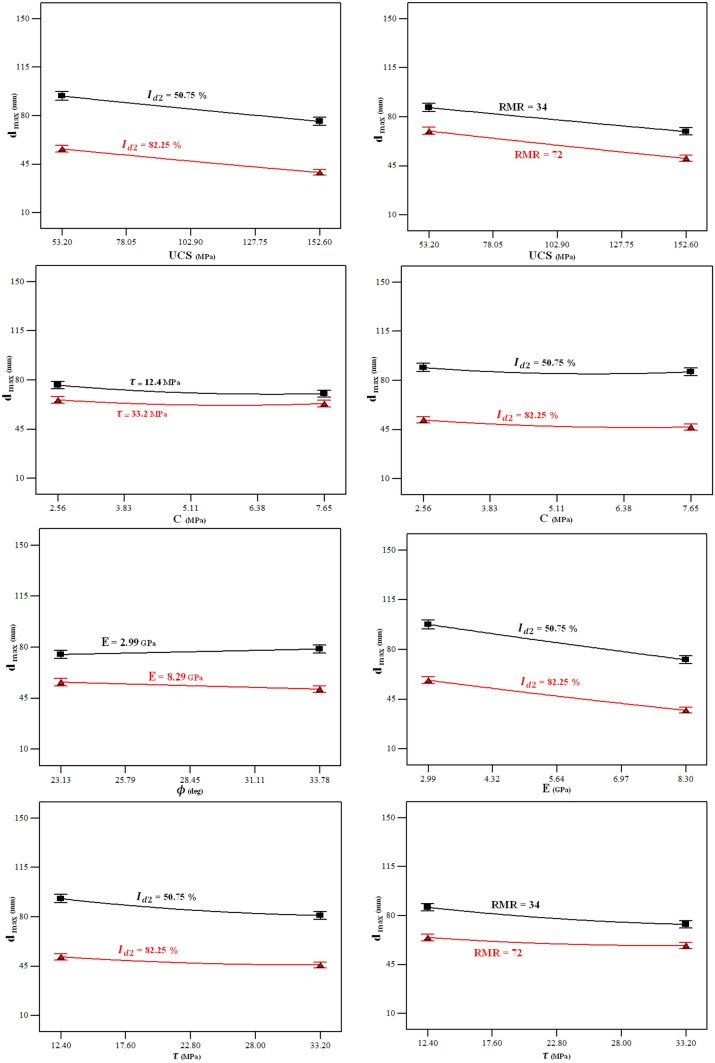
Pairwise interaction effects of input variables on the ${d}_{max}$.

In addition, the ANOVA results show that there are meaningful pairwise interaction effects between $UCS$ and ${I}_{d2}$, $UCS$ and $RMR$, $C$ and $\tau$, $C$ and ${I}_{d2}$, $\phi$ and $E$, $E$ and ${I}_{d2}$, $\tau$ and ${I}_{d2}$, and $\tau$ and $RMR$. Figure 13 illustrates the pairwise interactions between these input variables, and their effect on the ${d}_{max}$.

**Figure 14:**
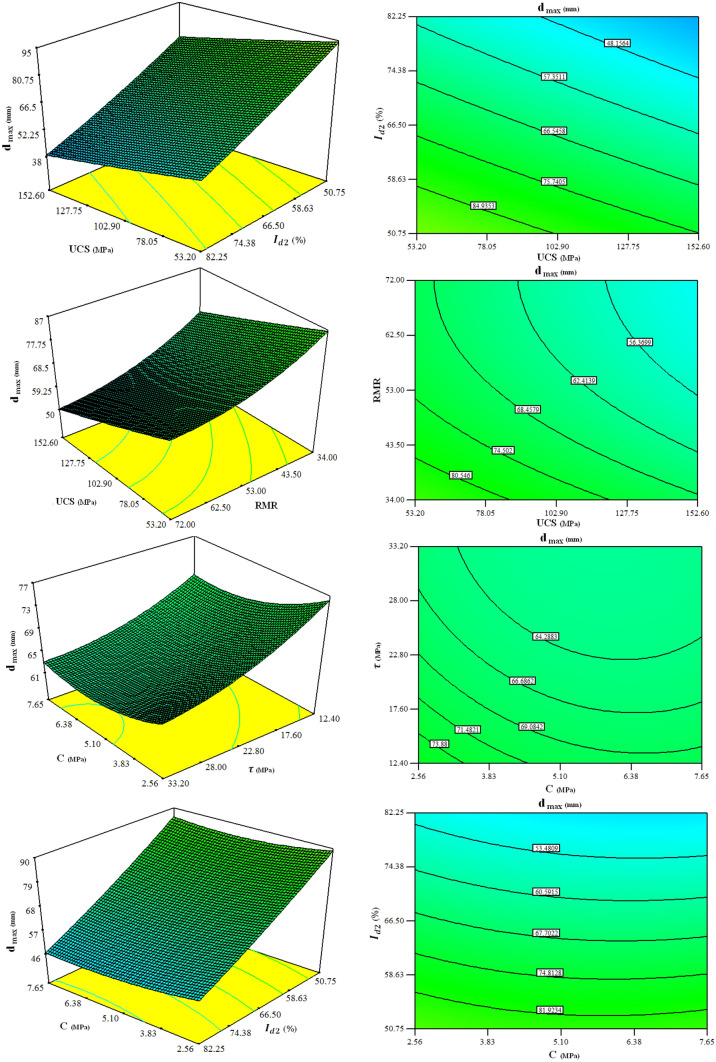

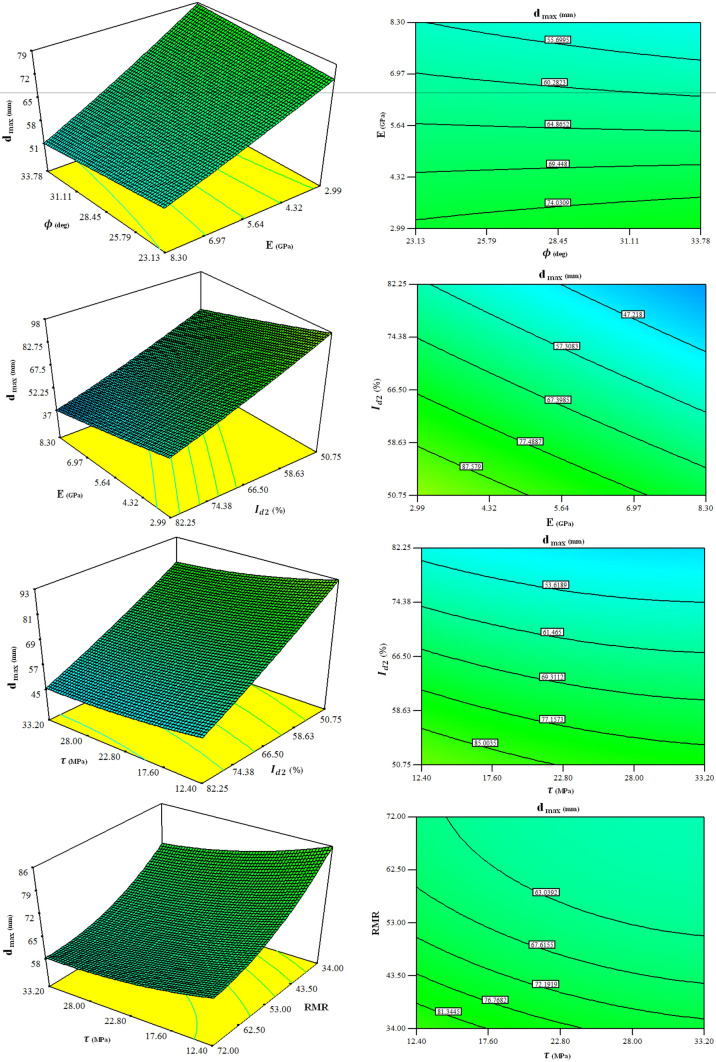
3D response surface plots showing interaction effects of variables on the ${d}_{max}$.

In order to further recognize the interactions between input parameters and their effect on the ${d}_{max}$, the 3D response surface plots, and contour plots are also drawn in Fig. 14 based on the coefficients of the proposed model, and the ANOVA results.

### Prediction capability of the proposed model

Since the ${R}^{2}$ and ${R}_{adj}^{2}$ indicators are unable to provide enough information for the prediction capability of the model, the ${R}_{pre}^{2}$ is used in our research which could prosperously detect the overfitting phenomenon in the cubic model. Moreover, the prediction capabilities of the models are examined by testing all models using unseen actual data that were monitored at the mine. Hence, in order to evaluate the validity of the investigated models, the results are compared with the real values of ${d}_{max}$.

**Figure 15:**
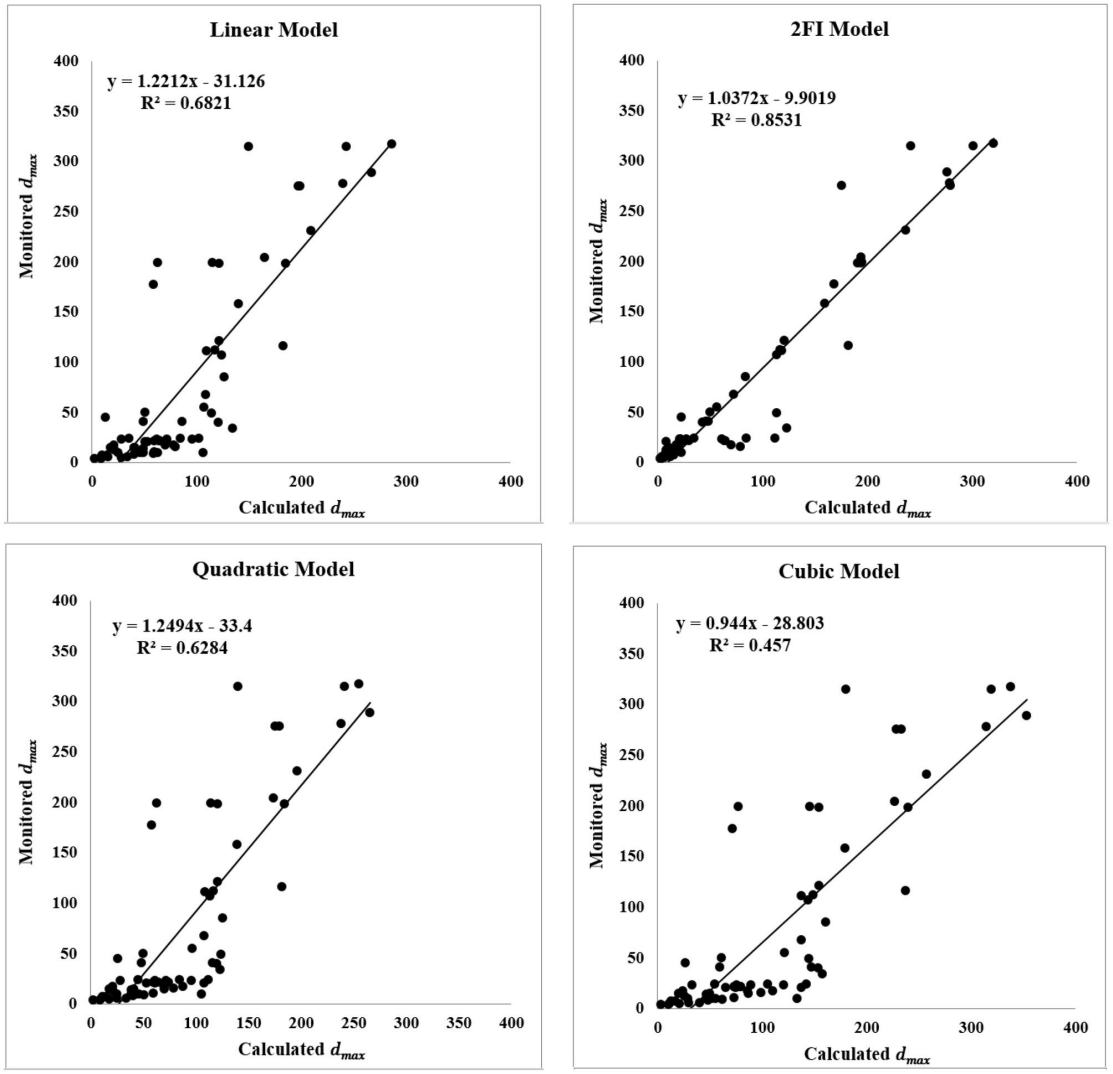
${R}^{2}$ derived from testing on unseen actual data for linear, 2FI, quadratic and cubic models.

**Figure 16:**
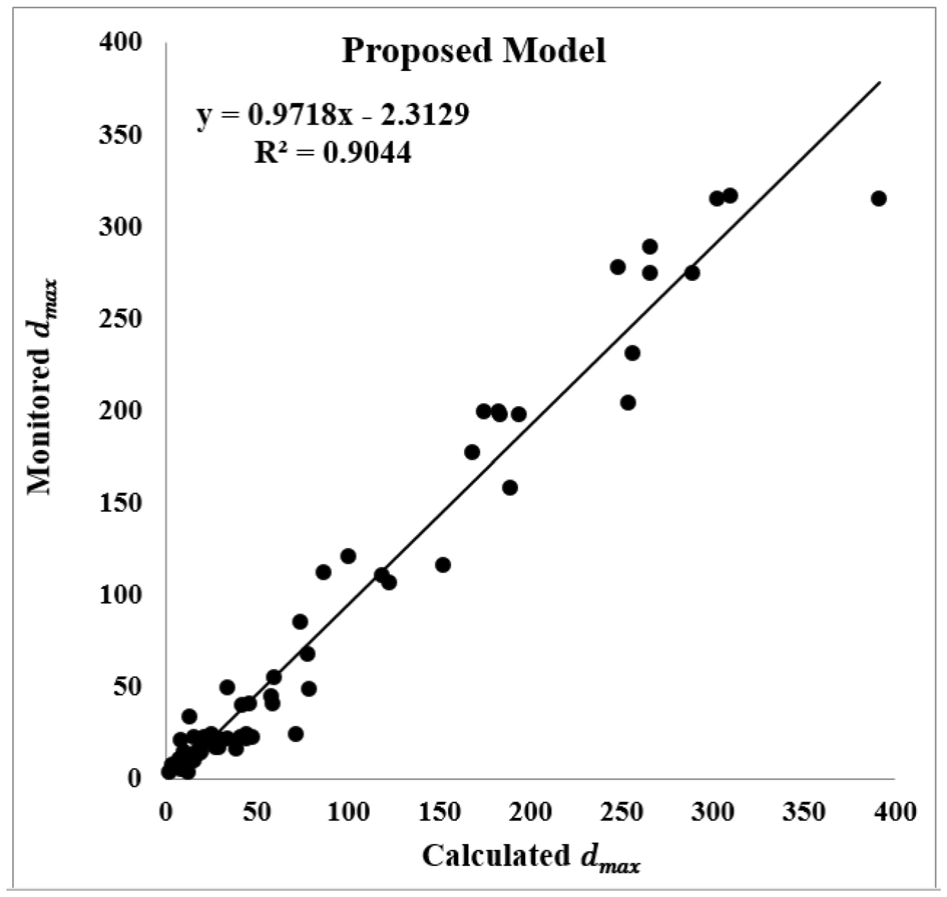
${R}^{2}$ derived from testing the proposed model by unseen actual data.

The real values of ${d}_{max}$ were obtained by monitoring the behavior of the tailgate roadway using dual-height telltales. The ${d}_{max}$ monitored in 68 sections are introduced to the proposed model, and the results are compared with the linear, 2FI, quadratic, and cubic models. The ${R}^{2}$ for four models of the linear, 2FI, quadratic, and cubic are respectively obtained as 0.68, 0.85, 0.63, and 0.46, which are presented in Fig. 15. The calculated ${R}^{2}$ for the proposed model is also obtained as 0.90 as shown in Fig. 16. As it can be seen, the coefficient of determination for the proposed model is of priority in relation to the other ones.

**Figure 17:**
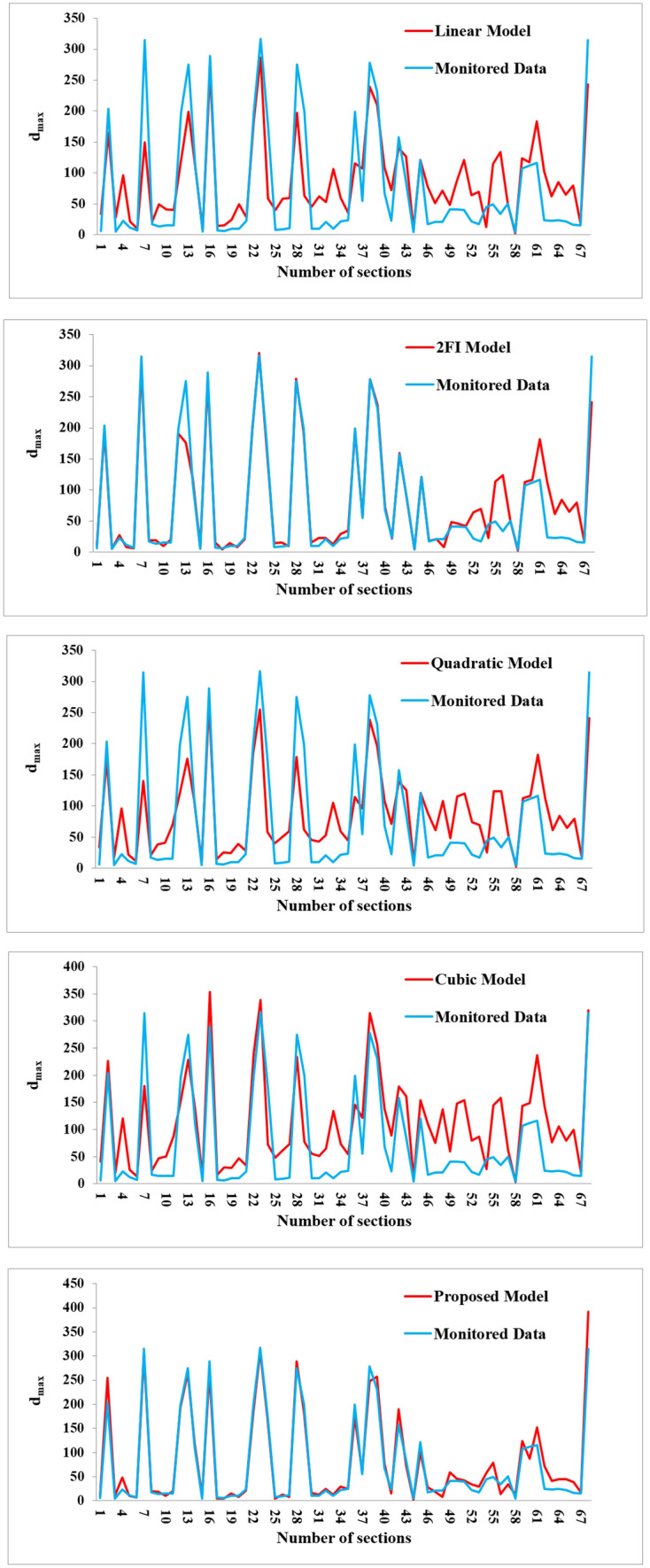
Comparing the results of all models with the real monitored data in 68 sections.

The results of the calculated ${d}_{max}$ obtained by the linear, 2FI, quadratic, cubic, and the proposed models are compared with the real ${d}_{max}$ measured from the roof displacement monitoring program at the mine. These results are compared with the real monitored data in 68 sections in Fig. 17, showing the priority and prediction capability of the proposed model when encountering with unseen data.

Support system optimization in longwall roadways is more important than ever due to reducing the mining costs, and ensuring the safety of the mine’s personnel. There are currently no methods that logically provide mine engineers with reliable arranges of support systems in longwall gate roadways especially in the tailgate roadway. The reliance on experience or at worst trial and error for support systems designing in longwall coal mining is a major contributing factor responsible for mine downtime and potential calamitous consequences^9,41^.

Due to the fact that the ground reaction behavior is a function of support load density and stiffness of the support system^42^, measuring roof displacements is a practical and rational measure to indicate the unstable zones, and control the potential instabilities through support system optimization.

The proposed model is an approach to optimize support systems and also to indicate the most affecting factor on the roof displacement as well as to investigate the interaction between independent variables. Nonetheless, a suitable DOE is to be achieved for rational optimization of support systems installed at the mine. The proposed model may be useful to avoid the difficulties associated with trial and error or the complexity of further time-consuming numerical simulations.

Therefore, based on the ${d}_{max}$ value obtained by the proposed model, the support systems will be optimized for controlling the deformation to a predetermined level. Accordingly, a margin of safety is provided, which reduces the risk of roof failure without necessity to excessive roof support.

Employing the finite-difference based RSM, it is possible to develop a suitable experimental design, which integrates the dominant independent variables and to present a reliable equation to estimate the response value based on a well-designed regression analysis. The results of the proposed model can be graphically represented as 3D response surface plots and contour plots, which can be assisted to describe the effects of the natural variables on the response, and also provide information for pairwise interaction effects of variables.

Since wrong appraisal of the capacity and locality of the designed support systems in tailgate roadway may result in excessive roof displacements, and consequently roof failures which are accompanied by irrecoverable disasters in longwall mining^2,11^, designing a suitable support system in longwall tailgates is one of the major tasks in the mine design procedure. Therefore, employing the proposed model may assist engineers to ensure that the tailgate roadway is functional. Based on the results obtained from the proposed model, it is possible to install a set of standing support systems ahead of time to control the immediate roof in the vicinity of the unstable zones having high displacements. Also, it is feasible to determine the more sensitive geomechanical parameters, and to indicate the pairwise interaction effects of the geomechanical parameters on the roof displacements. Then, the support systems will be redesigned to be optimized based on the geological and geomechanical information to provide a safe and secure working face at underground.

According to our findings at the panel E2 of Tabas mine, the slake durability index, Young’s modulus, uniaxial compressive strength, and rock mass rating are respectively found to be more sensitive parameters that have dominant effects on the roof displacements in the tailgate roadway. Also, the ${I}_{d2}$ appears to be the most effective parameters on the ${d}_{max}$ in our case study, which emphasizes on the disintegration characteristics of the weak and clay-bearing rock strata at the mine. Hence, one of the findings of this research is to underscore the measurement of the weathering resistance of roof rocks at Tabas mine, which are mainly siltstones, mudstones, argillites, and other clay-bearing rocks. In addition, the ${d}_{max}$ will be decreased provided that the ${I}_{d2}$ enhances through reducing the standup time of exposed roof span and avoiding weathering.

## Conclusions

Problematic tailgate instabilities in Parvadeh coalfield are recognized as a serious concern which may cause to consequences varying from production delays to potentially injuries or fatalities and catastrophic failures with loss of the mine. The tailgate roadway serves two roles in the adjacent longwall panels; firstly as a headgate for the previous panel, and secondly as a tailgate for the current panel. Therefore, the tailgate roadway in a longwall panel endures a high-stress concentration. This research was conducted in the panel E2 at Tabas mine to predict the unstable zones in the tailgate roadway, and consequently optimize the support systems’ density. A finite-difference based RSM was employed to develop a new applicable equation for predicting roof strata displacement in the tailgate roadway, and also optimizing the support systems’ designing process at Tabas mine. A five-level nine-variable CCD was adopted to design 149 runs with an appropriate combination of the independent variables of $\rho$, $UCS$, $E$, $C$, $\phi$, $\tau$, ${\sigma }_{t}$, ${I}_{d2}$, and $RMR$. Thereafter, the ${d}_{max}$ for each experiment was estimated for the whole possible conditions through some FDM numerical simulations in FLAC software. Investigating various linear, 2FI, quadratic, and cubic models, a modified quadratic equation is developed to predict the ${d}_{max}$ through statistical analyses. The validity of the proposed model is fulfilled by calculating the ${R}_{adj}^{2}$, ${R}_{pre}^{2}$, MSE, and PRESS between the models’ outputs and the actual roof displacements. While ${R}^{2}$ for four models of the linear, 2FI, quadratic, and cubic are respectively obtained as 0.68, 0.85, 0.63, and 0.46, the proposed model appears to give a high goodness of fit with an accuracy of 0.90, which reveals the prediction proficiency of the proposed model in relation to the others. Unlike the cubic and quadratic models, in our proposed model the value of 0.9258 for ${R}_{pre}^{2}$ is in reasonable agreement with a value of 0.9532 for ${R}_{adj}^{2}$, and avoids overfitting. Therefore, the proposed model may be applied as a reliable tool to estimate the roof displacement in the longwall tailgate without further complicated and time-consuming numerical simulations. ANOVA is implemented on the results of the proposed model to investigate the effect of each input parameter on the ${d}_{max}$, and also investigate their pairwise interactions. The calculated F-value and *p* value for lack of fit are respectively 3.66 and 0.2388, which may imply that the proposed model is satisfactory, and the lack of fit is not significant in relation to the pure error. The resulted VIF values are also in the range of unity, showing a supreme situation for the proposed model. In addition, the parameters ${I}_{d2}$, $E$, $UCS$, and $RMR$ are the key parameters influencing the ${d}_{max}$, while the parameters $\phi$ and $\rho$ do not have a significant effect on the response variable. Furthermore, there are meaningful pairwise interaction effects between $UCS$ and ${I}_{d2}$, $UCS$ and $RMR$, $C$ and $\tau$, $C$ and ${I}_{d2}$, $\phi$ and $E$, $E$ and ${I}_{d2}$, $\tau$ and ${I}_{d2}$, and $\tau$ and $RMR$. Amongst them, the ${I}_{d2}$ appears to be the most effective input variable on the ${d}_{max}$, showing the role of the weak and clay-bearing rock strata on the tailgate’s stability. In addition, the ${d}_{max}$ will be decreased provided that the ${I}_{d2}$ enhances through reducing the standup time of exposed roof span and avoiding weathering.
